# Parathyroid Hormone (PTH) Increases Skeletal Tumour Growth and Alters Tumour Distribution in an In Vivo Model of Breast Cancer

**DOI:** 10.3390/ijms19102920

**Published:** 2018-09-26

**Authors:** Hannah K. Brown, Gloria Allocca, Penelope D. Ottewell, Ning Wang, Nicola J. Brown, Peter I. Croucher, Colby L. Eaton, Ingunn Holen

**Affiliations:** 1 Department of Oncology and Metabolism, Mellanby Centre for Bone Research, University of Sheffield, Sheffield S10 2RX, UK; gallocca1@sheffield.ac.uk (G.A.); p.d.ottewell@sheffield.ac.uk (P.D.O.); N.Wang@sheffield.ac.uk (N.W.); N.J.Brown@sheffield.ac.uk (N.J.B.); c.l.eaton@sheffield.ac.uk (C.L.E.); I.Holen@Sheffield.ac.uk (I.H.); 2 Bone Biology Division, Garvan Institute of Medical Research, Sydney, NSW 2010, Australia; p.croucher@garvan.org.au

**Keywords:** parathyroid hormone, osteoblast, bone metastasis, breast cancer, bone metastatic niche

## Abstract

Breast cancer cells colonize the skeleton by homing to specific niches, but the involvement of osteoblasts in tumour cell seeding, colonization, and progression is unknown. We used an in vivo model to determine how increasing the number of cells of the osteoblast lineage with parathyroid hormone (PTH) modified subsequent skeletal colonization by breast cancer cells. BALB/c nude mice were injected for five consecutive days with PBS (control) or PTH and then injected with DiD-labelled breast cancer cells via the intra-cardiac route. Effects of PTH on the bone microenvironment and tumour cell colonization and growth was analyzed using bioluminescence imaging, two-photon microscopy, and histological analysis. PTH treatment caused a significant, transient increase in osteoblast numbers compared to control, whereas bone volume/structure in the tibia was unaffected. There were no differences in the number of tumour cells seeding to the tibias, or in the number of tumours in the hind legs, between the control and PTH group. However, animals pre-treated with PTH had a significantly higher number of tumour colonies distributed throughout skeletal sites outside the hind limbs. This is the first demonstration that PTH-induced stimulation of osteoblastic cells may result in alternative skeletal sites becoming available for breast cancer cell colonization.

## 1. Introduction

The majority of patients with advanced breast cancer will develop bone metastases [1], and there is emerging evidence that tumour cells disseminate to bone at very early stages of the disease [2]. Disseminated tumour cells (DTCs) can remain dormant within the bone marrow for many years before being triggered to proliferate [3]. The nature of these trigger(s) are currently not fully understood, but recent research demonstrated that osteoclast-mediated bone loss induced through either ovariectomy or castration can trigger growth of DTCs in bone in models of breast [4] and prostate cancer [5], respectively. Furthermore, in models of myeloma, specific activation of osteoclasts by treatment with the soluble ligand for receptor activator of NFkB (sRANKL) decreases dormant cancer cell numbers and promotes tumour growth [6]. A more comprehensive understanding of the crosstalk between tumour cells and the bone microenvironment in the very early steps in tumour arrival and the initiation of growth in bone is required in order to effectively target (and ultimately prevent) bone metastases. DTCs engraft into specific areas within the bone, and these putative metastatic niches comprise of several cell types, including bone cells, cells of the vasculature and the hematopoietic system. The areas of bone commonly colonized by tumour cells contain a number of proposed specialist niches, which are at least partly overlapping, e.g., the endosteal niche [7], the peri-vascular niche [8], and the hematopoietic stem cell (HSC) niche [9]. This suggests that the biological signals regulating hematopoiesis, vascular growth/renewal and bone turnover potentially converge on the same set of structures, the functions of which are interconnected. For example, Shiozawa et al. demonstrated that in models of prostate cancer, tumour cells compete with HSCs for space in bone niches [9]. We and others have recently reported that breast tumour cells appear to home preferentially in osteoblast rich areas of bone [7,10], and osteoblastic cells are in turn shown to regulate the HSC niche [11] as well as being closely linked with vascular remodeling in bone [12]. These observations indicate that osteoblasts are important components of the bone metastatic niche, but their contribution in supporting tumour cell engraftment, dormancy and survival remains to be defined. Establishing the role of the osteoblast in bone metastasis may be achieved either through depletion or stimulation of osteoblastic cell populations in vivo, followed by monitoring of tumour cell colonization of the resulting modified bone niche. Whilst depletion of osteoblasts in bone is challenging and likely to cause more widespread effects, intermittent PTH treatment leads to expansion of the osteoblastic compartment in bone, resulting in an anabolic effect by combined increased osteoblast number and activity [13,14,15,16]. Administration of PTH may modify tumour cells themselves, precluding identification of the specific effects on the osteoblastic niche. In order to assess the influence of the osteoblastic niche on tumour cell engraftment, we designed specific in vivo studies to establish whether PTH-mediated expansion of the osteoblastic compartment, prior to tumour cell injection, affects subsequent establishment of breast tumour cell residency or lesion formation in bone. By combining state of the art imaging of disseminated tumour cells and bone histomorphometry, we provide evidence that increasing osteoblastic cell number/activity results in increased breast tumour growth at multiple skeletal sites. We tracked tumour cells in vivo by staining them prior to injection with the fluorescent dye, DiD, which allowed us to identify single dormant tumour cells as they arrived in bone and monitored the growth of lesions by their constitutive expression of luciferase and red fluorescence as populations expanded. Our data suggest that osteoblasts may be key components of the bone metastatic niche and hence represent a potential therapeutic target. The results may also have implications for the use of PTH as a bone anabolic agent in the cancer setting and highlights the need to define the specific role(s) of osteoblasts in the development of bone metastasis in human disease.

## 2. Results

### 2.1. Short-Term Intermittent PTH Treatment Increased Osteoblast Numbers and Activity

**Figure 1:**
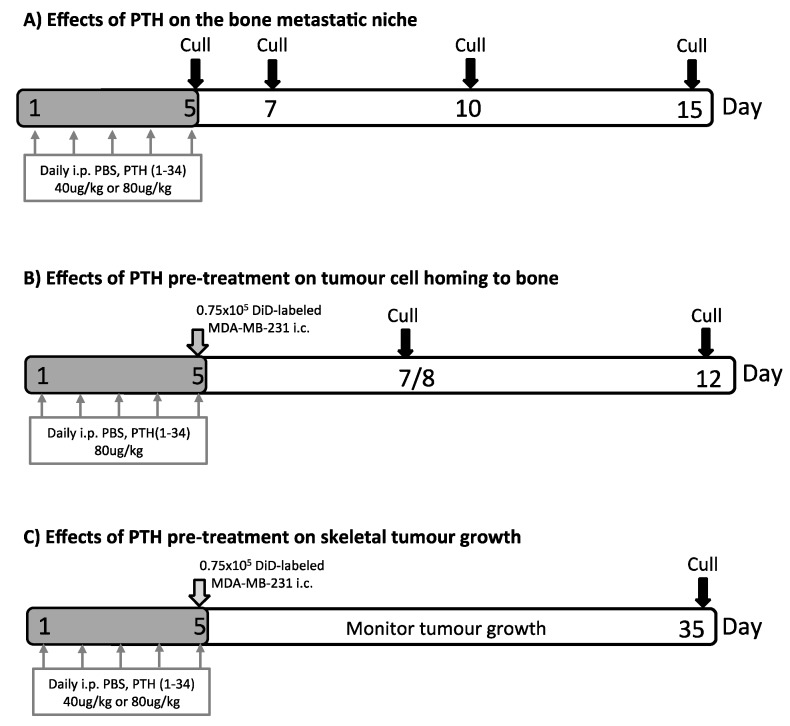
Experimental outlines. Twelve-week-old female BALB/c nude mice were used in all experiments. Mice were injected with PBS, 40 μg/kg or 80 μg/kg PTH daily for 5 days as indicated. In (**A**) effects of PTH treatment on bone volume, serum bone remodeling markers and bone cell numbers were determined. (**B**) To assess tumour cell homing to the long bones, animals were injected with 0.75 × 10^5^ DiD-labeled MDA-MB-231-tomato-luc2 cells 4 h after the last PTH injection on day 5. Samples were collected on day 7/8 and 12 before analysis of DiD-positive events in the bone marrow of the proximal tibia using two-photon microscopy. (**C**) Animals were treated with PTH and injected with tumour cells as described in (**B**). Investigation of effects of PTH pre-treatment on MDA-MB-231-tomato-luc2 tumour growth was performed using in vivo bioluminescence imaging and histology.

**Figure 2:**
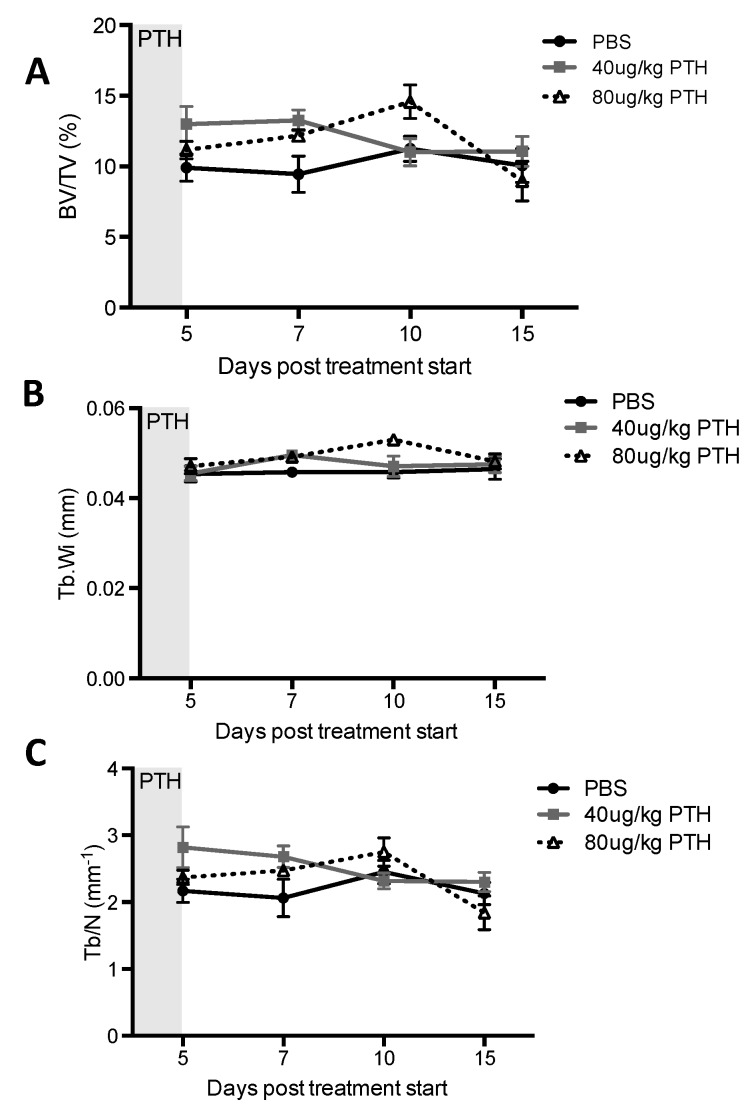
Effects of intermittent PTH on bone remodeling in 12-week old BALB/c nude mice. μCT analysis was performed on the proximal tibia of mice treated with 40 μg/kg or 80 μg/kg PTH or PBS for 5 days and mice were culled on day 5, 7, 10 and 15. Graphs show measurements of (**A**) trabecular bone volume per tissue volume (expressed as % BV/TV), (**B**) trabecular width (Tb.Wi, mm) and (**C**) trabecular number (Tb/N per mm). Data is presented as mean ± SEM and statistical analysis was performed using two-way ANOVA and multiple comparison post-test. A minimum of *n* = 5 animals per time point and treatment group were analyzed.

**Figure 3:**
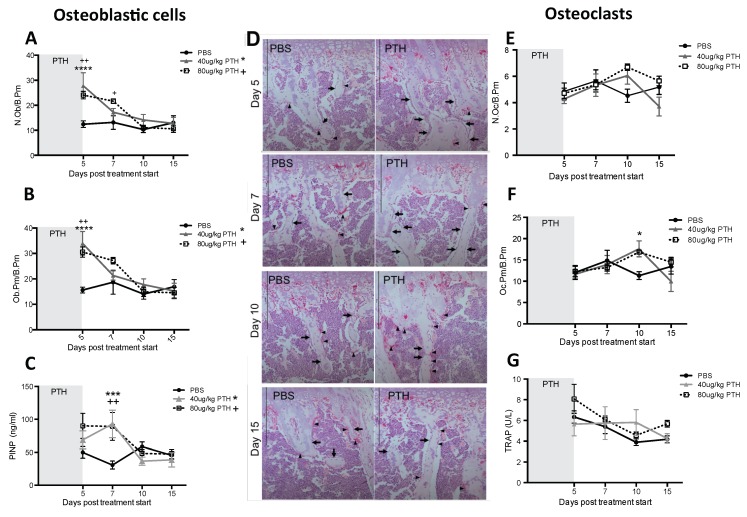
Intermittent PTH treatment induces changes to the bone microenvironment. Animals were treated with 40 μg/kg or 80 μg/kg PTH or PBS for 5 days and culled on day 5, 7, 10 and 15. TRAP stained histological sections of tibias were used to assess. (**A**) the number of osteoblasts per mm trabecular bone surface (N.Ob/B.Pm) and (**B**) the percentage of trabecular bone in contact with osteoblasts (Ob.Pm/B.Pm). (**C**) Serum was used for the analysis of the bone formation marker PINP by ELISA. (**D**) Representative TRAP images of sections from PTH and PBS treated mice. Osteoclasts are stained in bright pink (black arrowhead). Arrows show rows of osteoblastic cells. Scale bar = 100 μm. (**E**) Osteoclasts per mm trabecular bone surface (N.Oc/B.Pm) and (**F**) percentage of osteoclasts in contact with trabecular bone surfaces (Oc.Pm/B.Pm) were scored on histological sections stained for TRAP. (**G**) Serum marker of bone resorption (TRAP) was measured by ELISA. Data is presented as mean ± SEM and statistical analysis was performed using two-way ANOVA and multiple comparison post-test. * is 40 μg/kg PTH vs. PBS and * is *p* < 0.05, *** is *p* < 0.001 and **** is *p* < 0.0001. + is 80 μg/kg PTH vs. PBS and ++ is *p* < 0.01. A minimum of *n* = 4 animals per time point and treatment group were analyzed.

**Figure 4:**
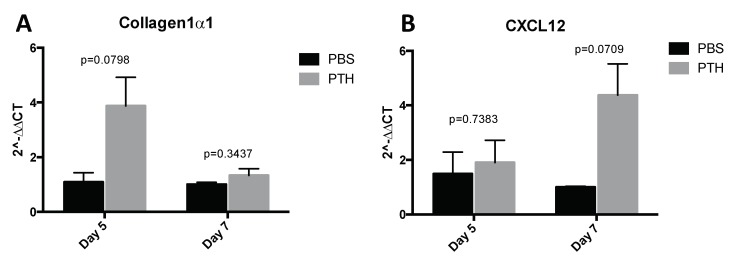
Assessment of osteoblast related gene expression in the front legs after PTH treatment. Mice were treated with 80 μg/kg PTH (*n* = 4–5) or PBS control (*n* = 3) for 5 days and culled on day 5 and day 7. RNA was extracted from whole front legs and qPCR for detection of the osteoblast related molecules Col1α1 and CXCL12 was performed. All data normalized to GAPDH. Data are presented as the mean ± SEM. Statistical analysis by unpaired students *t*-test, *p* values are indicated in the graph.

Our first aim was to identify a PTH dosing regimen that would affect osteoblasts in our model system, but not osteoclasts or bone volume and structure. We therefore performed a detailed longitudinal study to establish the effects of short-term (5 days) treatment with two doses (40 and 80 μg/kg) of PTH on bone remodeling parameters in 12-week old female BALBc/nude mice (see experimental outline in Figure 1A). We analyzed the metaphysis areas of tibiae, the regions of these bones most commonly colonized by breast tumour cells in this model. Compared to the controls, there was no significant effect of PTH on trabecular bone volume (BV/TV, Figure 2A), width (Tb.Wi, Figure 2B) or number (Tb/N, Figure 2C), as measured by μCT up to day 15. In contrast, PTH treated animals had significantly increased numbers of osteoblasts/mm bone surface (N.Ob/B.Pm day 5: 40 μg PTH vs. control: 27.80 vs. 12.14, *p* < 0.0001. 80 μg/kg PTH vs. control: 24.07 vs. 12.14, *p* < 0.01 and on day 7: 21.64 vs. 13.17, *p* < 0.05, Figure 3A) as well as percentage of bone in contact with osteoblasts in the trabecular bone of the proximal tibia (Ob.Pm/B.Pm day 5: 40 μg/kg PTH vs. control: 33.91 vs. 15.59, *p* < 0.0001 and 80 μg/kg PTH vs. control 30.56 vs. 15.59, *p* < 0.01, Figure 3B). This was accompanied by a significant increase in the serum level of the bone formation marker PINP on day 7 (40 μg/kg PTH vs. control: 92.94 vs. 30.87, *p* < 0.001 and 80 μg/kg PTH vs. control: 89.31 vs. 30.87, *p* < 0.01, Figure 3C). Examples of trabecular bone areas from tibia from control and PTH treated animals at each time point with osteoblasts and TRAP stained osteoclasts indicated are shown in Figure 3D. The PTH effects on osteoblasts were not restricted to the hind limbs. PCR analyses of osteoblast-related genes demonstrated an increase in expression of collagen 1α (day 5) and CXCL12 (day 7) compared to control, in the front limbs of animals treated with 80 μg/kg PTH for 5 days (Figure 4). The number of TRAP positive osteoclasts/mm trabecular bone surface (N.Oc/B.Pm) was not significantly different between PTH and PBS treated mice (Figure 3E). The only effect of PTH detected on osteoclasts was an increase in the percentage of bone in contact with osteoclasts in animals treated with 40 μg/kg PTH on day 10 (Oc.Pm/B.Pm: 17.62 vs. 11.30, *p* < 0.05, Figure 3F), with this parameter returning to control levels by day 15. The serum level of the bone resorption marker TRAP5b did not differ between PTH and PBS treated animals at any time point, supporting that osteoclasts were largely unaffected by this treatment schedule (Figure 3G). For all other analyses, there was no significant difference between the effects of 40 and 80 μg/kg PTH and all effects of PTH were normalized to control levels by day 15.

These results confirmed that intermittent PTH treatment for five consecutive days predominantly induces changes to osteoblastic cells, increasing their number on trabecular bone surfaces of the tibia as well as their activity in the long bones. 

### 2.2. PTH Does Not Modify Tumour Cell Seeding to the Hind Limbs

**Figure 5:**
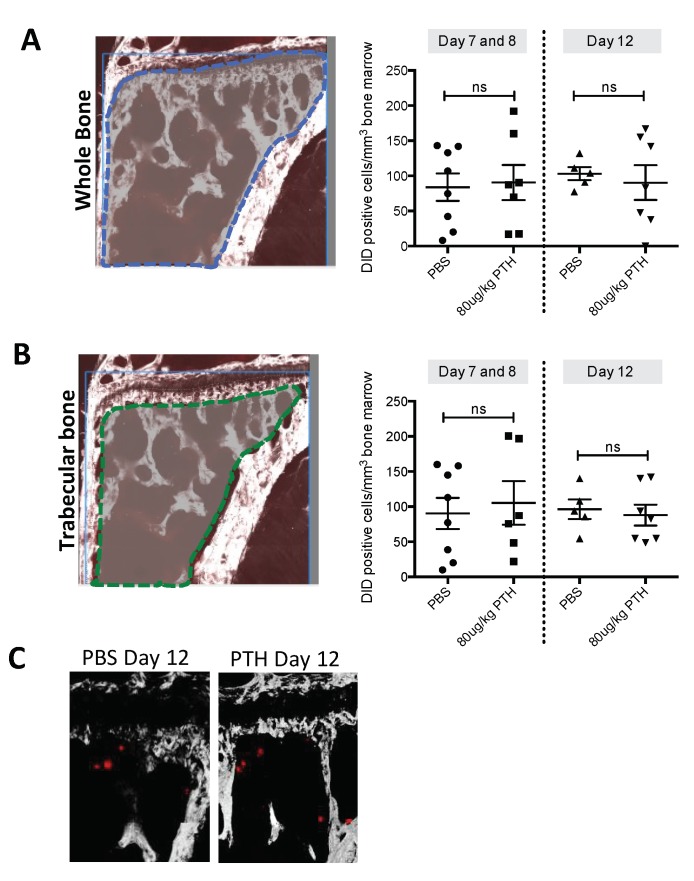
PTH pre-treatment does not alter tumour cell seeding to long bones in vivo. Twelve-week-old female BALB/c nude mice were treated with 80 μg/kg PTH or PBS before injection of DiD-labelled tumour cells. Animals were culled on day 7/8 and 12 and tibias were prepared for two-photon microscopy. (**A**) A stack area of 2104 μm × 2525 μm below the growth plate and 70 μm depth was visualized using a 633 nm laser to detect DiD stained tumour cells (referred to as DiD-positive events). (**B**) The same analysis as in A but including only the trabecular bone area. Data is presented as mean ± SEM and statistical analysis was performed using *t*-test. No significant difference was detected. A minimum of *n* = 5 tibias per group were analyzed. (**C**) Example images of DiD-positive events (red) in the tibia imaged by multiphoton microscopy (bone: white, imaged using second harmonic generation).

Having established that osteoblastic cells were found in a higher density and in a more activated state with increased activation in PTH treated mice compared to control, we hypothesized that this could potentially expand the osteoblastic niche, resulting in an increased number of tumour cells homing to bone. To test this, 12-week-old female mice were treated with PBS or 80 μg/kg PTH for 5 days before injection of DiD-labelled MDA-MB-231-td tomato-luc2 tumour cells on day 5 into the left cardiac ventricle (see outline in Figure 1B). PTH has a short half-life in vivo and was cleared from the circulation prior to tumour cell injection. Animals were culled on day 7/8 or 12, and tumour cell numbers in the proximal tibia assessed by two-photon microscopy. We found that pre-treatment with PTH did not alter the number of DiD-positive cells detected in either the trabecular bone area (Day 7/8: PBS 90.31 vs. PTH 105.33. Day 12: PBS 96.42 vs. 94.62; Figure 5B) or in a larger region including the trabecular, cortical bone and the growth plate area (Day 7/8: PBS 83.79 vs. PTH 90.60. Day 12: PBS 103.04 vs. PTH 90.37; Figure 5A). Figure 5C indicates examples of DiD positive events in bone imaged by multiphoton microscopy. These data suggest that the PTH-induced alteration of the osteoblastic component of the bone microenvironment does not affect the number of tumour cells seeding to the hind limbs. 

### 2.3. Pre-Treatment with PTH Modifies Number and Distribution of Breast Tumours in Bone

**Figure 6:**
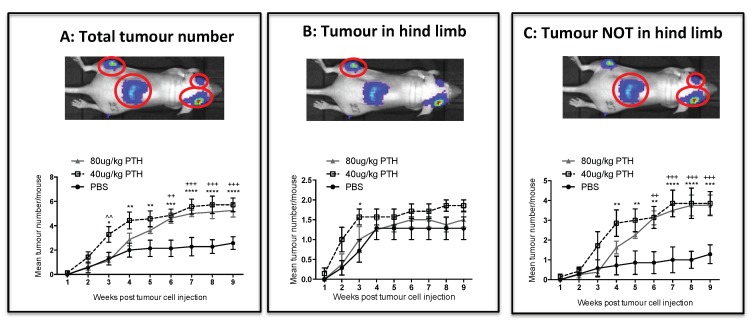
PTH pre-treatment affects development of tumours in an in vivo bone metastasis model. Twelve- week-old female BALB/c nude mice were treated with 40 μg/kg, 80 μg/kg PTH or PBS daily for 5 days before injection of tumour cells and monitoring of tumour growth by bioluminescence imaging. The number of detectable luciferase positive tumours was recorded for each mouse and is shown in three categories including: (**A**) the total number of tumours, (**B**) tumours detected in hind legs and (**C**) tumours detected in all sites except the hind legs. Data is presented as mean ± SEM and statistical analysis was performed using matched two-way ANOVA and multiple comparison post-test. * is 40 μg/kg PTH vs. PBS, ^ is 40 μg/kg PTH vs. 80 μg/kg PTH, + is 80 μg/kg PTH vs. PBS., A minimum of *n* = 6 animals per time point and treatment group were analyzed.

**Figure 7:**
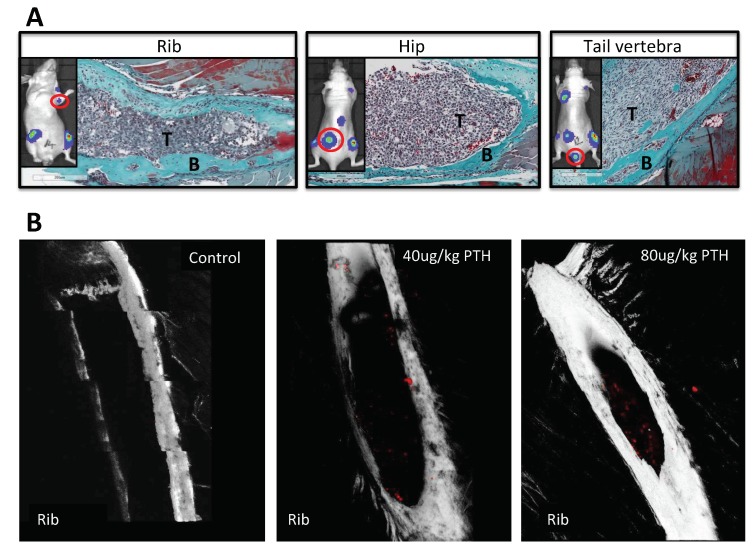
PTH increases tumour growth in skeletal sites outside the hind limbs. (**A**) Representative images of histological slides from tumours (indicated by T) detected in rib, hip and tail following Goldner’s trichrome staining to highlight bone (in green, indicated by B) from animals pre-treated with PTH. The inserts show the corresponding bioluminescence image for each site with the presented tumour colony highlighted by a red circle. (**B**) Example images obtained by 2-photon microscopy of ribs isolated from animals treated with PBS, 40 μg/kg or 80 μg/kg PTH for five days prior to injection of 0.75 × 10^5^ DiD-labeled MDA-MB-231-tomato-luc2 cells. A stack area of 1262 μm × 1683 μm at 70 μm depth was visualized using a 633 nm laser to detect DiD labelled tumour cells (in red).

**Figure A1:**
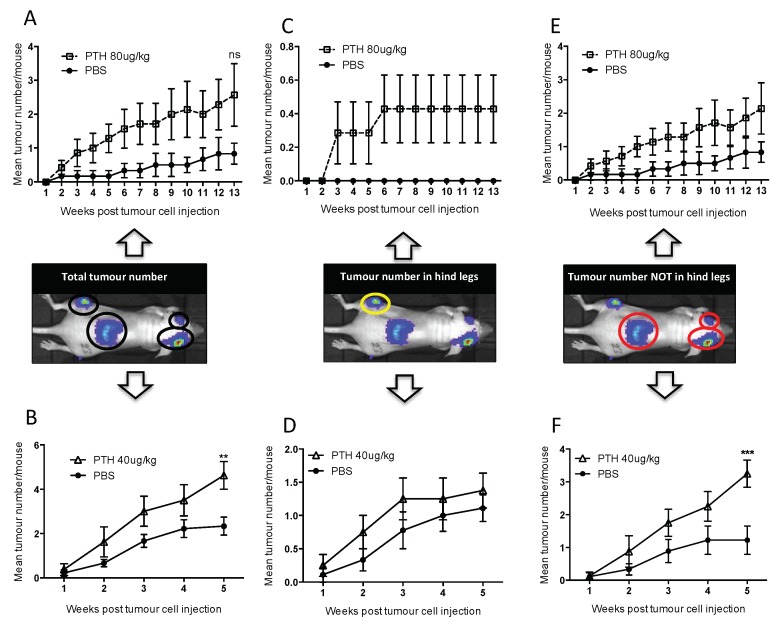
Tumour distribution at different skeletal sites. Twelve-week-old female BALB/c nude mice were treated with 80 μg/kg PTH or PBS (**A**,**C**,**E**) and 40 μg/kg PTH or PBS (**B**,**D**,**F**) before injection of DiD-labeled MDA-MB-231-tomato-luc2 cells and monitoring of tumour growth by in vivo bioluminescence imaging. The number of detectable luciferase positive tumours was recorded for each mouse and is shown in three categories including (**A**,**B**) the total number of tumours, (**C**,**D**) tumours detected in hind legs and (**E**,**F**) tumours detected in all other sites except the hind legs. Data is presented as mean tumour number/mouse ± SEM and statistical analysis was performed using matched two-way ANOVA and multiple comparison post-test. * is 40 μg/kg PTH vs. PBS and ** is *p* < 0.01 and *** is *p* < 0.001.

We next investigated whether pre-treatment of animals with PTH affected the development of tumours in vivo (see outline in Figure 1C). To examine this, breast cancer cells were injected into animals at time points where we had determined osteoblastic cell numbers would be increased in response to treatment with PTH (Figure 3A–C). Twelve-week-old female BALB/c nude mice were treated for 5 days with PBS, 40 or 80 μg/kg PTH, before intracardiac injection of MDA-MB-231-dt tomato-luc2 cells 3–4 h after the last PTH administration on day 5. Tumour growth was monitored for the following nine weeks. Crucially, as PTH is cleared within 2–3 h [15], and animals did not receive further PTH treatment after tumour cell injection, this design allowed us to assess the effects of PTH on the microenvironment, but not on the tumour cells themselves. We performed longitudinal in vivo bioluminescence imaging and determined the numbers and location of tumours present over time using this technique in three separate experiments. Lesions were also assessed post-mortem at the end of experiments by ex-vivo fluorescence and histology. The intracardiac model used in this study has been shown to result in development of tumours predominantly in the hind legs; however, in PTH treated animals, luciferase positive tumour colonies were also frequently detected in other skeletal sites, most notably the front legs and the ribs (Table 1, Appendix A, Figure A1). To determine the effects of PTH on tumour growth we analyzed tumour number per mouse in three different ways: (1) Total tumour number; (2) tumours only detected in the hind legs; and (3) tumours detected in sites other than the hind legs (Figure 6, Table 1). Animals that were pre-treated with PTH for 5 days prior to tumour cell injection had significantly higher total numbers of skeletal tumours compared to animals receiving PBS (Figure 6A, mean numbers of tumours/mouse: 40 μg/kg PTH: 5.71 vs. control: 2.57, *p* < 0.0001 and 80 μg/kg PTH: 5.25 vs. control 2.57, *p* < 0.001). The tumour burden in the hind legs was very similar in all groups (Figure 6B). In contrast, there were significantly higher numbers of tumours detected in other sites in PTH-treated mice vs. control (Figure 6C, mean numbers of tumours/mouse in 40 μg/kg PTH: 3.86 vs. control: 1.29, *p* < 0.001, 80 μg/kg PTH: 3.75 vs. control: 1.29, *p* < 0.001). Histological analysis of luc2+ cells within tissues verified that tumours outside the long bones were still associated with the skeleton, including in the ribs, hip, and tail (Figure 7A). We analyzed a limited number of ribs (*n* = 3 control and *n* = 3 PTH) by 2-photon microscopy to determine if tumour cell homing differed at this site. We found no DiD-labelled tumour cells in the ribs from the control animals (see example image in Figure 7B), in agreement with the lack of tumours detected (Table 1). In contrast, although the sample size was small, there were abundant DiD-labelled cells present in all ribs examined in the PTH-treated animals (Figure 7B). These results require further confirmation in a larger sample set, but appear to suggest that PTH-induced alteration of the bone microenvironment increases tumour cell homing to the ribs, but not to the hind limbs (Figure 5). Taken together, our data show that administration of PTH prior to tumour cell injection significantly increased the number of skeletal breast tumours compared to control, mainly in sites outside the hind limbs.

## 3. Discussion

The composition of the putative bone metastatic niches remains to be defined, but it appears in model systems that these structures are located in the same area of bone that contains vascular, osteoblastic and HSC niches. These niches may overlap, as they include many of the same cell types, making it challenging to attribute specific niche functions to particular cell populations. We aimed to elucidate the role of the osteoblast in the bone metastatic niche. The most significant novel finding we present is that increasing the osteoblastic cell population with PTH resulted in breast cancer cells subsequently arriving in this modified environment, forming significantly higher numbers of skeletal tumours outside the hind limbs compared to control. In addition, there was a differential distribution of skeletal tumours, with PTH-pretreated animals having tumours in ribs, hip, vertebrae, skull and front limbs, whereas control animals almost exclusively had tumours in the hind limbs. These data suggest that PTH-induced osteoblastogenesis results in increased availability of suitable skeletal sites for tumour cells to colonize.

We used the established bone anabolic agent rhPTH 1–34 to increase the number of osteoblastic cells in bone in vivo, which is cleared within a few hours of administration [15,17,18]. We developed a treatment schedule that significantly modified osteoblasts, but without altering the number and/or activity of the tightly coupled osteoclasts. This is of great importance, as increased osteoclast activity stimulates the growth of tumour cells in bone in this and other models [4,5,6,19]. Bone volume and structure was unaffected by PTH throughout our study; hence, the effects on tumour growth were not due to gross alterations of the microenvironment. In addition, the brief period of PTH treatment (five days) was completed prior to injection of tumour cells in the animals, thus avoiding any direct effects of PTH on tumour growth. Previous studies in mice have reported PTH-induced increases of osteoblastic cells as early as two days after initiation of treatment [20]. In agreement with this, we found significantly increased numbers of osteoblasts in the proximal tibia after five days of treatment with 40 or 80 μg/kg PTH, the earliest time point analyzed. PTH also increased expression of osteoblast-related genes in the front legs, showing that the effects were not limited to the tibia. Short-term PTH most likely mediated an increase in osteoblastic cells, mainly through inhibition of apoptosis, although increased differentiation of bone lining cells is also a suggested mechanism [14,18]. We have established that following intra-cardiac injection in BALBc/nude mice, breast cancer cells reach the bone microenvironment within 24 h (our unpublished data). As the lifespan of osteoblasts in mice is estimated to be around 12 days [21], their number would remain increased following PTH treatment during the key period of tumour cell homing and colonization of the bone metastatic niche in our model.

Our studies focused on characterizing effects on the osteoblastic niche, but PTH has also been shown to modify the HSC and vascular niches [22,23]. Work by Calvi et al. demonstrated that PTH is a regulator of the HSC microenvironment, and that pulse treatment with PTH caused HSC mobilization and hence increased survival in mice undergoing myeloablative bone marrow transplantations [11]. Subsequent studies by the same group found that administration of PTH augmented the effects of HSC mobilization using G-CSF and protected the HSCs from the effects of chemotherapy [24]. The results from these and other studies lead to clinical trials of PTH in support of HSC mobilization [22,25]. The implications of this approach for disseminated tumour cells in the bone marrow remains to be established, but elegant work by the Taichman group has shown that in models of prostate cancer, tumour cells compete with HSC for space in the niche [9]. In their models, administration of HSC mobilizing agents to tumour-bearing animals resulted in increased numbers of tumour cells in the circulation, but the effects on subsequent tumour growth was not determined. It is not known if breast cancer cells also compete for space in the HSC niche, or whether PTH causes mobilization of tumour cells that reside in HSC niches. It is important to note that the studies of HSC mobilization have all used much longer periods of PTH treatment (several weeks) than the five-day course given in the present study [22]. Although we did not monitor HSCs, simulation of this process is unlikely to have influenced our results, as we did not inject tumour cells until after the PTH treatment was completed. In addition, PTH did not appear to make space available for tumour cells in the HSC niche, as there was no increase in the number of tumour cells homing to the long bones in PTH treated animals, compared to control. 

**Figure A2:**
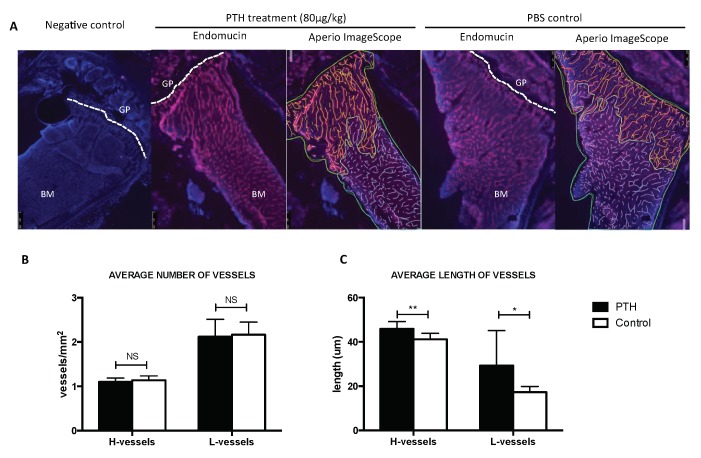
Effects of 80 μg/kg PTH on the bone microvasculature. (**A**) Tibia sections of animals from control and PTH treated animals (*n* = 5/group) were stained with antibody against the endothelial cell marker Endomucin (in red), nuclei were counterstained with DAPI (blue) (GP = growth plate, BM = bone marrow). Aperio ImageScope images show the quantification of the vessels performed manually (green = area of bone marrow analyzed, yellow = H-vessels, blue = L-vessels). Number of H- and L-vessels in both groups are shown in (**B**) while the average vessel length is shown in (**C**). Graphs show mean ± SEM, * *p* < 0.05 and ** *p* < 0.005 by 2 way ANOVA.

Osteogenesis and angiogenesis are closely coupled processes during bone remodeling, with PTH receptor signaling in osteoblasts shown to modulate both bone formation and vascularization of the growth plate [26]. There are few reports of the direct effects of PTH on bone vasculature, but a study in rats has reported a spatial redistribution of capillaries in the tibia caused by 15 days of PTH treatment [23]. Intriguingly, they found that intermittent PTH increased VEGF A gene expression but did not stimulate bone angiogenesis. Instead, PTH induced the relocation of smaller vessels in close proximity to sites of active bone formation, but the rate of microvascular redistribution was not assessed. This finding is of particular importance in light of the key role that the perivascular niche is suggested to play in the regulation of tumour cell dormancy, where recent elegant studies have demonstrated that the bone microvasculature induces sustained tumour cell quiescence, which is lost in areas of vascular sprouting [8]. By analyzing a small number of samples, we found that five days of PTH treatment modified the length, but not the number, of vessels in the metaphysis of the hind limbs (Appendix B Figure A2). This was not associated with increased tumour growth at this site. In future studies, we will establish the effects of short-term PTH treatment on both osteoblasts and the vasculature in different skeletal sites and map whether subsequent tumour cell homing is modulated. It is possible that PTH induces modification of the vascular niches outside the long bones, with implications for bone remodeling, tumour cell location, mobility and proliferation. 

The most intriguing finding in our study is that PTH appeared to exert differential effects on tumour growth depending on the skeletal site. While there was no difference in the tumour burden in the hind limbs, PTH treated animals had significantly increased tumour growth in the front legs, vertebrae, ribs and hip. This may be due to the variations in the bone microenvironment between the sites, including bone structure, composition and osteoblast content, resulting in a differential response to PTH. Previous studies in prostate cancer models have reported that in animals with subcutaneous tumours receiving a daily administration of PTH for three weeks, increased numbers of tumour cells were recovered from the mandible, rib, spine and tibia, but not from femurs, compared to control [27]. In agreement with our findings, these results support that PTH treatment can affect tumour cell seeding to a number of skeletal sites outside the long bones. The mechanism underlying this differential effect of PTH between sites was not identified, as the study design did not establish whether PTH affected the primary tumour, metastatic spread, or the bone microenvironment. Similarly, Schneider et al. showed that intermittent PTH treatment increased prostate cancer tumours in hind limbs and craniofacial regions compared to control [28]. In contrast to these findings, and to our data, is a recent study by Swami et al. reporting beneficial effects of PTH treatment on overall survival in breast cancer models, which was linked to reduced skeletal homing and growth in skeletal sites [29]. The discrepancies to our findings is likely explained by the different treatment regimens used, as the Swami study investigated the effects of long-term PTH treatment, which continued after tumour cells were injected, as well as use of different animal models (4T1 immunocompetent model used in the Swami study whereas we used an immunocompromised model). 

PTH has been shown to have different effects on cancellous compared to periosteal osteoblasts [14,30] and in vertebrae compared to femurs [31], but whether this also happens after short-term exposure warrants further investigation. Importantly we did establish that osteoblastic gene expression was upregulated in the front legs following PTH treatment, supporting that increased tumour growth in this area is linked to osteoblast number/activity. The present study is the first to show that intermittent PTH not only increases the number of tumour cells in different skeletal sites, but also results in increased numbers of overt tumours developing long after the PTH effects on bone have reverted to control levels. 

Our study has some limitations; we could not determine whether the increased tumour growth outside the hind legs was due to increased tumour cell homing, stimulation of DTCs to form overt tumour colonies, or a combination of both. In future experiments, we will map the presence of tumour cells in a variety of skeletal sites following intra-cardiac injection of DiD-labelled tumour cells. It is complex to quantify the number of osteoblasts in skeletal sites outside the long bones, and we were unable to directly compare the effects of PTH on comparable bone areas in samples from ribs or front limbs. 

PTH is approved as a treatment for osteoporosis, but not for cancer-induced bone disease or bone loss caused by anti-cancer therapies [32]. Due to studies showing that prolonged exposure to PTH caused osteosarcoma in rat models [33], there has been concern surrounding long-term effects in patients although there is no evidence that patients receiving PTH to treat osteoporosis are of increased risk of developing osteosarcoma [34]. However, there is increasing interest in the use of anabolic agents in the treatment of breast cancer bone metastases to improve bone health (reviewed by Chirgwin et al.) [35]. Whether our data have relevance for the clinical setting remains to be determined, but the bone microenvironment clearly plays a role in human bone metastasis progression, including in breast cancer [36]. The role of the osteoclast in this process is well established, whereas that of the osteoblast is largely unknown. Our data support the notion that osteoblastic cells are essential for breast cancer tumour cell homing and growth in skeletal sites. Furthermore, the results highlight the importance for additional investigations into the use of PTH as an anabolic agent in the treatment of breast cancer bone metastases to gain a clearer understanding of the role of the osteoblast in metastatic disease.

## 4. Materials and Methods 

### 4.1. Cells

The MDA-MB-231-td-tomato-luc2 human breast cancer cell line was purchased from Caliper Life Sciences (Cheshire, UK) and maintained in RPMI-1640 (Gibco, Thermo Fisher Scientific, Loughborough, UK) supplemented with 10% fetal bovine serum (Sigma Aldrich, Gillingham, UK). 

### 4.2. Animals

Twelve-week-old female BALB/c nude immunocompromised mice (Charles River, UK) were used in all procedures. Groups of 4–6 animals were housed in a controlled environment in Optimice cages (Animal Care Systems, Centennial, CO, USA) with a 12-h light/dark cycle at 22 °C with ad libitum 2918 Teklad Global 18% protein rodent irradiated diet containing 1.01% calcium (Harlan Laboratories, Derby, UK) and water. Husbandry conditions were specific pathogen free (University of Sheffield) and adhered to the Home Office Code of Practice https://www.gov.uk/government/uploads/system/uploads/attachment_data/file/388535/CoPanimalsWeb.pdf. All procedures complied with the UK Animals (Scientific Procedures) Act 1986 and were reviewed and approved by the local Research Ethics Committees of the University of Sheffield under Home Office project license 40/3462 (Sheffield, UK).

### 4.3. PTH Treatment and Intracardiac Injection of Tumour Cells

Mice were injected intraperitoneally with PBS or rhPTH 1–34 (Bachem, Bubendorf, Switzerland) at 40 μg/kg or 80 μg/kg on five consecutive days [17]. For the assessment of bone-remodeling parameters, animals were culled on day 5, 7, 10 and 15 (Figure 1A). For long-term tumour experiments, PTH treatment was carried out as above. On day 5, (4 h after administration of the last PTH dose) MDA-MB-231-td-tomato-luc2 cells were stained with the lipophilic dye DiD (1,1′-Dioctadecyl-′, 3′-Tetramethylindodicarbocyanine, 4-Chlorobenzenesulfonate, Vybrant DiD cell labelling solution, Thermo Fischer) before preparing a single cell suspension in PBS. 0.75 × 10^5^ DiD-labelled MDA-MB-231-td-tomato-luc2 cells were injected into the left cardiac ventricle of mice under isofluorane anesthesia and tumour growth monitored in vivo by bioluminescence imaging for the duration of the experiments using the IVIS Lumina II (Caliper Life Sciences, Preston Brook, UK). Intra-cardiac injection of this MDA-MB-231 clone mainly results in tumour growth in the hind limbs with occasional tumours in vertebrae, skull, jaw and adrenal glands, whereas other soft tissues are unaffected (see tumour distribution in the control group, Table 1). An outline of the tumour experiments is shown in Figure 1B,C. Serum was collected and stored at −80 °C, bones for multiphoton microscopy were snap frozen in liquid nitrogen and bones for histological assessment were fixed in 4% PFA for 48 h prior to decalcification (0.5M EDTA, 0.5% PFA, PBS, pH 8) for 2 weeks followed by infiltration with paraffin. 

### 4.4. Two-Photon Microscopy

Dissected snap frozen tibias and ribs were embedded in Cryo-M-Bed (Bright Instrument Co. Ltd., Huntingdon, UK) and trimmed longitudinally to expose the bone marrow using a cryostat (Bright Instrument Co. Ltd.). The bones were fixed to a glass bottom dish and visualized using the Zeiss LSM510 NLO two-photon microscope (Carl Zeiss Ltd, Cambridge, UK). A stack area of 1262 μm × 1683 μm for the ribs and 2104 μm × 2525 μm for the tibias both at 70 μm depth was visualized using a 633 nm laser and BP 650–710 filter to detect DiD labelled tumour cells (referred to as DiD-positive events). Bone matrix was visualized using the two photon laser (Coherent, Santa Clara, CA, USA) set at 900 nm and a BP 390–465 filter. Volocity 3D Image Analysis software 6.01 (PerkinElmer, Seer Green, UK) was used to quantify the number of DiD-positive events. 

### 4.5. Preparation of Samples for Analyses of the Bone Microvasculature

Hind limbs from control and PTH-treated animals (*n* = 5/group) were fixed in 4% ice-cold PFA for 4 h and decalcified in 0.5M EDTA at 4 °C for 24 h. Decalcified bones were subsequently immersed in ice- cold CPT solution (20% Sucrose and 2% PVP prepared in PBS) for 24 h. Bones were transferred to EMB solution (8% gelatin, 20% sucrose and 2% PVP prepared in PBS), warmed to 60 °C in water bath for 45 mins and then placed in molds to set at ambient temperature. The embedded samples were stored at −80 °C and 30 μm sections cut with a cryostat (Microm HM 560, Thermo Fisher Scientific). Cryosections were air dried prior to rehydration with PBS and permeabilization (0.3% Triton X-100 for 20 mins) followed by blocking in 5% normal goat serum (NGS, Vector Laboratories, Peterborough, UK) for 30 mins at ambient temperature. The primary antibody (Endomucin, sc-65495, Santa Cruz) was diluted 1:100 in 5% NGS and added to the bone sections for 1 h at ambient temperature. Sections were washed with PBS and incubated with the secondary antibody (Alexa-fluor 555, A21434, Thermo Fisher Scientific) diluted 1:200 in PBS for 1 h at ambient temperature. Nuclei were counterstained with VECTASHIELD^®^ Mounting Medium with DAPI.

### 4.6. Quantification of the Effects on the Microvasculature

An inverted widefield fluorescence microscope (AF6000, Leica, Milton Keynes, UK) was used to acquire images of the immofluorescent staining. Microvessels positively stained for Endomucin were manually tracked using Aperio ImageScope software. The shape of the capillaries was used to identify the different types of vessels: column-like ducts were assumed to be H-vessels while highly branched sinusoids were considered L-vessels [12]. Analyses were performed on three non-consecutive levels of both tibias of each animal (*n* = 5/group, 30 sections quantified in total).

### 4.7. Serum Bone Remodelling Markers Measured by ELISA

Serum levels of tartrate-resistant acid phosphatase (TRAP) and N-terminal propeptide of type I procollagen (PINP) ELISA were used to quantify bone resorption and formation activity, respectively (both from Immunodiagnostic Systems, Tyne and Wear, UK). Whole blood was collected and serum was extracted after centrifugation. Serum aliquots were stored at −80 °C until use. Both ELISAs were performed according to manufacturers’ instructions.

### 4.8. Quantitative Real Time PCR

Front legs were stripped of muscle and bones were snap frozen in liquid nitrogen before storage at −80 °C. RNA was extracted from snap frozen front legs using TRIZOL according to the manufacturers protocol. cDNA synthesis was carried out using SuperScript III First Strand Synthesis SuperMix and qPCR was performed using TaqMan Universal PCR Master Mix and an ABI 7900 PCR System (Perkin Elmer, Foster City, CA, USA). Mouse collagen 1α1 and CXCL12 gene expression levels were analyzed using the following TaqMan probes: Col1α1 (Mm00801666_g1) and CXCL12 (Mm00445552_m1). All expression values were calculated using the 2^−∆∆*C*T^ method and normalized to GapdH (all reagents from Applied Biosystems, Warrington, UK).

### 4.9. Histological Quantification of Osteoclasts and Osteoblasts 

Visualization of bone structure was done on 3 μm thick histological sections of decalcified bone following Masson-Goldner trichrome staining kit (Merck, Watford, UK), performed according to the manufacturers’ instructions. Tartrate resistant acid phosphatase (TRAP) staining of osteoclasts (3 μm) and identification of osteoblasts using morphological criteria on 3 μm thick histological sections were performed as previously described [37]. The number of osteoblasts (N.Ob/B.Pm), osteoblast surface (Ob.Pm/B.Pm), the number of osteoclasts (N.Oc/B.Pm), and the osteoclast surface (Oc.Pm/B.Pm) were determined on two non-serial sections using a Leica RMRB upright microscope with a 10× objective and OsteoMeasure software (Osteometrics, Decatur, GA, USA). Bone cell numbers per mm/trabecular bone was determined on all trabecular surfaces 125 μm away from the growth plate. All histomorphometric parameters were based on the report of the ASBMR Histomorphometry nomenclature and performed in a blinded fashion [38].

### 4.10. Microcomputed Tomography 

Microcomputed tomography analysis of proximal tibias was performed using a Skyscan 1172 X-ray-computed microtomograph (Bruker, Kontich, Belgium) in accordance with the ASBMR guidelines [39]. Imaging was carried out at a voltage of 50 kV and a currency of 200 μA with a medium camera resolution of 2000 × 1024, an aluminium filter of 0.5 mm and pixel size was set to a dimension of 4.3 μm. Scanning was initiated from the proximal tibia. For each sample, images were reconstructed with NRecon software. The volume of interest (VOI) was then specified by interactively drawing on the two-dimensional acquisition images. For trabecular bone measurements, the VOI was composed only of cancellous bone, and the cortices were excluded. Trabecular bone volume (BV/TV in %), number (Tb/N per mm-1) and width (Tb/Wi, mm) of tibias were calculated from the lowest part of the growth plate and covering 1 mm of the bone.

### 4.11. Statistical Analysis

Prism 6 software (GraphPad, La Jolla, CA, USA) was used for all statistical analysis. Unpaired *t*-test, one-way ANOVA with Tukey post-test, two-way ANOVA or matched two-way ANOVA with Tukey or Dunnet’s post-test were used. For quantification of the microvasculature and of DiD positive events, Students *t*-test was used. In all cases, a *p*-value of *p* ≤ 0.05 was considered to be significant. 

## Figures and Tables

**Table 1:** Overview of tumour distribution in PTH pre-treated animals. Overview of tumour distribution in skeletal and non-skeletal sites in animals treated with 40 μg/kg or 80 μg/kg PTH or PBS for 5 days followed by injection of DiD-labeled MDA-MB-231-tomato-luc2 cells. The number of detectable luciferase positive tumours was recorded for each mouse and the location is indicated in the table for hind limbs, front limbs, ribs, kidney and other sites. The mean tumour number/mouse in each group is indicated from one of three representative experiments.

Group	Mouse	Hind Limbs	Adrenal Gland	Front Limbs	Ribs	Other Sites	Total no of Tumours	Tumours/Mouse
**Control**	1	Both	Left	--	--	--	3	
	2	Right	Right	--	--	--	2	
	3	Right	--	Both	--	--	3	
	5	Both	Right	Both	--	--	5	
	7	--	--	--	--	Head	2	
	8	Right	--	--	--	--	1	
	***N* = 6**	**5/6**	**3/6**	**2/6**	**0/6**	**1/6**	**16**	**2.66**
**40 µg/kg PTH**	9	Both	Right	Left	--	Head	5	
	10	Both	Both	Left	Right	--	6	
	12	Both	Both	Left	--	Tail, spine	7	
	13	Right	Both	Left	Left	Jaw	6	
	14	Both	--	Left	Right	--	4	
	15	Both	Right	?	?	Head	4	
	16	Both	Right	--	Right	Head	5	
	***N* = 7**	**7/7**	**6/7**	**5/7**	**4/7**	**5/7**	**37**	**5.28**
**80 µg/kg PTH**	17	Both	--	--	Left	--	3	
	18	Left	Left	--	Right	--	3	
	19	Both	Both	Left	--	Hip /pelvis	6	
	20	Left	Right	--	--	--	2	
	21	Left	Left	--	--	--	2	
	22	Left	Both	--	Left	--	4	
	23	Left	Left	--	--	Head	>3	
	24	Both	Both	--	Left	--	5	
	***N* = 8**	**8/8**	**7/8**	**1/8**	**4/8**	**2/8**	**28**	**3.50**

## Abbreviations

DTC	Disseminated Tumour Cell
PTH	Parathyroid Hormone
Ob	Osteoblast
Oc	Osteoclast
HSC	Hematopoietic Stem Cell
uCT	Microcomputed Tomography
